# The English Government Hits Limits: Knowledge Politics and Covid-19

**DOI:** 10.3389/fsoc.2021.684658

**Published:** 2021-07-22

**Authors:** Tim May

**Affiliations:** ^1^ Faculty of Social Sciences and Department of Sociological Studies, University of Sheffield, Sheffield, United Kingdom; ^2^ Faculty of Social Sciences, The University of Sheffield, Sheffield, United Kingdom

**Keywords:** politics, knowledge, pandemic, Covid-19, science, policy, judgement

## Abstract

Tragic its consequences, the Covid-19 pandemic has ripped through societies with, at the time of writing, global death rates exceeding two and a half million people. In the process, there has been variability in terms of how effectively governments have dealt with the pandemic. Differences between political responses, forms of governance and the relationships between science and politics are apparent. This article investigates these relations in the United Kingdom with a particular focus upon the interpretations that informed the response of the English Government and their interactions with the scientific community. For this purpose, it provides an exploration of the political and socio-economic conditions prevailing in the United Kingdom prior to the pandemic. It then examines the interactions between science and politics as the pandemic unfolded during 2020. Then, building on these discussions it views the tensions that arose through a clash between two characteristics within democratic societies: the redemptive and pragmatic. What becomes apparent is the tendency for a form of the redemptive to be favoured over the pragmatic which results in an exposure of limits to usual and narrow political ways of governing.

## Introduction

The Covid-19 pandemic has ripped through societies. At the time of writing, global death rates are estimated to have exceeded three million people with clear differences in the effectiveness of how governments have handled the pandemic. The purpose of this article is to investigate the United Kingdom with a particular focus upon the English Government, the political climate informing their responses and their interactions with the scientific community.

The article is divided into three sections. First, an exploration of the social and political and economic conditions prevailing in the United Kingdom prior to the pandemic that inform the subsequent responses. Second, an examination of the interactions between science and politics as the pandemic unfolded during 2020. In the interactions between the credibility of scientific knowledge and its political interpretation and applicability, a greater understanding of the responses and their consequences emerge. Third, an analysis of these dynamics that draws upon discussions from the first part of the article and views the tensions that arose through a clash between two characteristics within democratic societies.

These insights are influenced by developments in sociological theory. In its refusal to see a simple distinction between the social and economic, it takes inspiration from classical social theory and in particular, the work of Max Weber, who once described himself as a “social economist” (Holton and Turner, 1990). In its examination of socio-economic conditions, prior to the pandemic, it draws upon writings on individualization and fragmentation and the role of the public sphere in society, along with the sociology of knowledge and science. That latter influence is apparent in its examination of the dynamics between science and politics during the pandemic with an emphasis upon the role of legitimacy in public expectations of political decisions and scientific advice. To further understanding of these dynamics during the pandemic, it turns to political sociology and writings on populism to chart the content and limits to what have been exposed as narrow ways of governing society.

## Pre-Pandemic Social Conditions: Understanding Responses

We have seen an expansion in our horizons of meaning over the course of the pandemic. In the United Kingdom normal political practices have become ones in which risk is individualized and citizens are expected both to be flexible and to exhibit sufficient commitment in the labour market (Dardot and Laval 2013). It is an era in which doubt is argued to have been seeded and perpetuated in the population, whilst the sphere of public, political accountability shrinks (McGoey 2019). Informing this is a populism that constitutes “external” forces as threats to what are assumed to be traditional ways of life.

A focus on external threats has enabled politicians who extol these ways of being to speak of “taking back” control of national affairs to re-establish internal and sovereign matters. This is a populist challenge to political normalization that draws upon: “a reservoir of raw anti-status-quo feelings” (Laclau 2007). For this to make sense presupposes the existence of established boundaries with the most prominent being the “national.” Promulgating alleged threats to liberty and property, alongside references to national cultures, goes to the core of the anxieties of citizens living in situations of socio-economic uncertainty. With the political sphere increasingly emptied of responsibility for ameliorating socio-economic conditions, the effect is to reduce concerns with inequality to matters of meritocracy; from there, a path to individual responsibility for the circumstances in which people live is forged.

These transformations have clear effects upon public and private realms in society. As the private realm is increasingly separated from a diminishing public sphere, the gaze of citizens shifts to their “own performance and thus diverted from the social space where the contradictions of individual existence are collectively produced.” The demand then arises: “for individual pegs upon which frightened individuals can collectively hang their individual fears” (Bauman 2001). The public sphere becomes less one of democratic concerns and the establishment of rights and more a “giant screen on which private worries are projected” (Bauman 2001). Accompanying this is the departure of power into an extraterritorial space of electronic networks and transnational financial practices which know no borders, leaving solace for those left behind to be sought in a myriad of opportunities for “escape, avoidance, disengagement and invisibility” (Bauman 2001).

The libertarians informing these views seek a world flattened through the removal of barriers to the realisation of their ambitions. Freed from such constraints the practices they pursue have been characterised as consuming their “own background conditions of possibility. It is like a tiger that eats its own tail” (Fraser 2019). The consequences for others include the production of huge inequalities, whilst resistance is countered by concerted efforts to place these visions beyond doubt through a process of agnotology. This is not: “the study of ignorance and doubt under all their manifestations, as sometimes mistakenly asserted, but rather the focused study of the intentional manufacture of doubt and uncertainty in the general populace for specific political motives” (Mirowski 2014). Think-tanks and consultancies, fed by the academic wares of university workers hungry for recognition, perpetuate these ways of seeing.

In the process a knowledge politics is born bolstered through a link to notions of “being” in society. Flexibility and uncertainty in the labour market combine with the prevention of forms of action through doubt informed by a suspicion of motives beyond anything other than self-interest. The terrain of the future, which the technicians of transformation who inhabit its territory are busy producing in the present, is rendered increasingly devoid of democratic participation (Mirowski 2014). An increasing absence of political accountability tends to remove contestation and relations between the economy and citizens are fed by anxiety and personal responsibility abstracted from social context. Forms of value then appear that categorize the “unworthy.” The consequence is a “virus of precarity” that runs alongside the rhetorical perpetuation of apparently limitless possibility in an unbounded marketplace (Lorey 2015).

In the shrinking space of political accountability comes the realm of blame, particularly of those who perform a public service: for example, care workers, nurses, doctors and teachers. At the start of the pandemic, the United Kingdom. National Health Service were estimated to be short of ten thousand doctors and forty thousand nurses (Davis 2021). Pre-pandemic tendencies were to denigrate and under value the idea of public service, whilst the responsibilities of Government were increasingly assumed to be limited through their efforts to naturalize “the economy.” These exercises of societal reconfiguration run in parallel to politicians positioning themselves as the defenders of liberty. Not surprisingly, this does not concern those socio-economic conditions which enable a citizen to participate in society, but a domain for the individual and their significant others which: “entails not simply the absence of frustration (which may be obtained by killing desires), but the absence of obstacles to possible choices and activities—the absence of obstructions on roads along which a man (sic) can decide to walk” (Berlin 1979).

As this occurs, rhetorical allegiances to the nation-state are accompanied by economic liberalization whereby we see the constant movement of capital across borders in order that the liberty of those with considerable funds are not stifled by oppressive tax regimes. The resulting funds tend to reproduce themselves and once established: “continue to grow at rapid pace for decades simply because of its size” (Piketty 2014). These extra-territorial ambitions are manifest in the property markets of global cities (Atkinson 2020), whilst the institutions which seek to regulate these actions require removal. As Jeffrey Alexander put it in his discussion of the 2008 financial crisis: “Democratic states stripped regulations off their economies like old paint peeling on a hot summer day” (Alexander, 2019: 40).

In these conditions governing is about the dismantling of barriers regarded as blockages to such ambitions. Government is assumed to have a minimal function: that is, to make up for market failures. Yet within this way of seeing the world, government failure easily replaces market failure (Friedman and Friedman, 1980). Overall, to curtail the reach of political power into the market requires: “repealing the regulatory state (while making the state itself the subject of regulation) and limiting the political voice of the people” (Brown 2019). Yet, to attribute simple homogeneity to elites in the production of this climate is problematic, whilst there are unintended consequences and circumstances, as we shall see, that temper their excesses.

In this respect we can detect different rationalities between the moral compass of the New Right and the amoral character of neo-liberal rationality. Nevertheless, this does not become a case of contradiction for there is an accord between the two. Whilst neo-liberal rationality promotes the idea of enterprise as residing within the exceptionality of an individual’s character divorced from social context, it is within the family where that morality is inculcated (see Dardot and Laval 2013). It is a different matter when it comes to those who funded Brexit in the City of London. The backers of “first wave financialization” favoured remaining in the European Union, whilst those in the “second wave” wanted to leave and provided significant resources for the Brexit campaign.

As Marlène Benquet and Théo Bourgeron (2021) characterize it, second wave financialization exhibits a mode of accumulation that encourages the public to save and take short-term decisions in the stock market with their managers having: “a largely passive management role and delegate the control of companies to business.” The second wave is concerned with asset management, hedge funds and private equity whose model of investment is: “only weakly correlated to the financial markets, either because their investments are unlisted or because they only invest in high-risk stock market sectors”. This latter group is concerned with what Sayer (2015) terms wealth extraction, not creation. It is their ambition to turn the City of London into a form of offshore investment platform and their influence can be traced into the heart of the United Kingdom. Government (see: https://mondediplo.com/2021/01/10uk).

The political face of those who pursue these practices are bolstered not just by those for whom these transformations are turned into technical matters apparently divorced from politics, but also those who appear to delight in the creation of turmoil and conflict. The uncertainty this generates operates within Government, as well as the financial and data mining sectors. As the Prime Minister’s Chief Adviser put in when seeking new recruits to such efforts, diversity is needed, but not that which is associated with race and gender. This was a reference to “cognitive diversity”: “We need some true wild cards … If you want to figure out what characters around Putin might do, or how international criminal gangs might exploit holes in our border security, you don’t want more Oxbridge English graduates who chat about Lacan at dinner parties with TV producers and spread fake news about fake news” (see: https://dominiccummings.com/2020/01/02/two-hands-are-a-lot-were-hiring-data-scientists-project-managers-policy-experts-assorted-weirdos/). After a series of clashes among powerful egos and a public focus during the pandemic on his own behaviour during lockdown, Cummings and the Director of Communications left Downing Street in November 2020 (see: https://www.bbc.co.uk/news/uk-politics-54938050).

These practices judge success according to narrow aspirations fed by a juggling act involving the permanent possibilities assumed to be contained in neo-liberal reproduction. Working not just at a macro, strategic level, but a tactical, micro-political level, they lock-in the direction of public policy through more subtle “capillary tactics” that seek to neutralize politics (Chamayou 2021). They have an insatiable appetite to re-fashion context and include those technicians of transformation who tinker: “with machines and models to demonstrate the mechanics of economic interaction” (Gibson-Graham, Cameron and Healy 2013). Accompanying this are centralised command and control models for the conception of public policy and the formulation of judgements as to whether it is being implemented effectively. Thus, despite the idea of a reduced role for governments, a strong state is needed to put markets in those places once occupied by the public sector. By re-directing demand into markets and away from alternative political aspirations the “big question of the choice of society can be evaded by dissolving it into the tiny questions of a society of choice” (Chamayou 2021: 231).

What about the knowledge produced for these purposes? What we find in these centralised modes is the tendency for public policy to separate the knowledge that it draws upon from the social and economic conditions under and through which it is enacted in given localities (May and Perry 2018). The content of knowledge becomes context-revising and certainly not sensitive, for that would mean recognition of existing conditions that detract from potentiality. Such a focus upon the future, accompanied by the idea of “lagging behind,” informs the frenetic reproduction of economic possibility. If this is challenged it becomes a matter of commitment to aspiration that is, minimally, indifferent to the present. In the absence of opposition to the conception of policy, the focus moves to the technicalities of implementation. The result is a triumph of process over purpose with the overall result that the relations between the “why” and “how” of knowledge are subsumed within the narrow confines of measurement (May, 2001).

With this comes a focus upon what is “relevant” and “useful.” Whilst these expectations of knowledge have always been with us, they have come to assume an increasing importance in circumscribing what knowledge should be produced and how it should be judged. Deliberations concerning “why” are displaced by the assumed technicalities of effective deployment: “talking about rational efficiency becomes a way of avoiding talking about what the efficiency is actually for: that is, the ultimately irrational aims that are assumed to be the ultimate ends of human behaviour” (Graeber 2015: 15). Calls for transparency in the motives of those who perpetuate and profit from these conditions are well made (O’Neil, 2016), but in the process knowledge becomes a tool of economic manipulation. Attempt to regulate, manage, control and direct produces a new form of “knowledge politics” (Stehr 1994) that blurs the domains between science, politics and the economy: science is linked to innovation for competitive advantage and wealth creation.

Scientific expertise has become more deeply integrated into the fabric of modern societies and manifest in decisions about the environment, health and welfare and security. Equally, business leaders can be scientists concerned with the technological implications of its insights and universities seek third stream funding from patents and spin out opportunities (May, 2018). As this occurs, politicians refer to the problems of expert control over everyday life. That leaves out critiques of the deployment of knowledge in politics and business activities and works to displace the importance of consideration of values outside of the narrow terrain created between the economy and its promise. Although the idea of a functioning market has been created by the ideas of economists, this works to frame and bound knowledge through an attributed value which denigrates alternatives and seeks to regulate what is credible and applicable (May and Perry, 2011).

These dynamics have effects upon public service and those infrastructures which provide security and can mitigate against the worst excesses of a pandemic. In pre-pandemic political rhetoric we found reference to the problem of democracy being governed by unelected and unaccountable expert bodies. That has a clear appeal. Democracy, after all, is a: “form of government that is based on the idea that no individual or any group has a title to govern over others” (Rancière 2010). However, a distrust and suspicion of such expertise can readily translate into disdain for public service, particularly if it does not conform to the idea of market rationality. The *Art of Judgement*, for instance, is an influential study on the complexity of public affairs and the process of policy making. Noting that there are debates over regulatory systems for political choices, between those who believe in the free market and executive agency, or a combination of those, Geoffrey Vickers writes: “It has always been necessary and desirable that the needs that it (the market) could not express or supply should be expressed and supplied politically” (Vickers, 1995: 155). That is a space of public service between politics and business, but one which now represents a direct challenge to knowledge and competences acquired outside of the market process.

In the United Kingdom public services have increasingly been required to reflect private business practices through the introduction of such things as internal and quasi markets. As this occurs, the Government outsources its expertise to consultancies and awards the private sector with sizeable contracts to perform what were once public services. The result is a shift in legitimacy for the competences informing a public service ethos and what remains of its practices are subject to market measures. What we see is not a liberal approach as expressed by Vickers, through the exercise of caution in respect to the strengths and limitations of markets, but an embrace of the influence of firms on the state (Crouch 2011). It can be cast as both inevitable and desirable, as if it were not the result of a chosen policy in the first place. As one architect of these changes wrote in a lecture during the pandemic entitled “*The Privilege of Public Service*,” more people in Government needed to be: “equipped to read a balance sheet and discuss what constitutes an appropriate return on investment” and to be “conversant with the commercial practices of those from whom we procure services and can negotiate the right contracts and enforce them appropriately” (Gove, M. https://www.gov.uk/government/speeches/the-privilege-of-public-service-given-as-the-ditchley-annual-lecture).

## Enter Science: Political Judgement in the Public Gaze

The limits of these forms of governing and deployment of selective knowledges were to be exposed during the pandemic. Political accountability had been avoided through speed of change, the creation of conditions of uncertainty and allusion to abstract, individual economic opportunity masquerading as public, collective possibility. Rancière’s (2006) two opposing systems of legitimacy in governmental authority were to collapse. First, there is the legitimacy sought through the popular vote and second, once elected, that which comes with the ability of political leaders to exercise judgement for the best possible solutions for societal problems.

Brexit is apparent in the first sense of legitimacy and toughness in respect to immigration and asylum policies are easy wins with respect to the second element. The latter are “external” threats that detract from “internal” issues less amenable to populist, rhetorical posturing. Covid-19, however, could not be externalised in this way. Denial or denunciation, as the first stage of reaction, becomes inadequate to the task of meeting a collective, public concern to act. So too is bravado and an almost aggressive nostalgia for the whole through allusion to collective belief bound within nation-states (whilst, it should be added, busily removing the borders which enable it). Ideas of “one nation” were also problematic because of the presence of the devolved administrations in the United Kingdom (Scotland, Wales and Northern Ireland) which enabled a differential response to the pandemic. Here was a societal sense of risk that translated into collective expectations of political leaders. It was born of a sense of fear as “the name we give to our *uncertainty*; to our *ignorance* of the threat and what is to be *done*” (Bauman 2006). These conditions were not simply about ignorance, but an absence of not knowing that translated into an expectation for political leaders to act in the public interest. The pandemic became a test of the second element of legitimacy. This was new.

The response started with what seemed to be an initial indifference and even denial on the part of political leaders. However, as the costs of political indecision mounted, the tendency to perpetuate an individualistic ethic was placed to one side as the United Kingdom entered its first lockdown in March 2020. Citizens were now encouraged to exhibit a relational responsibility in their actions: that is, to be concerned with and care for others, because we are all “in this together” as victims of a threat whose origins may have been external, but rapidly became internal. This call to collectivism was a test of politics as usual and would lead to ambiguity exhibited as an absence of clarity and coordination and willingness to learn from mistakes. That would have serious consequences for the British population. Disdain for public service and exacerbating precarity were not only inapplicable responses, but potentially disastrous ones. In the process media scrutiny heightened and even attempts by a core technician of turmoil to retrospectively position themselves as the predictor of pandemics would be exposed to public scrutiny (https://www.newstatesman.com/science-tech/2020/05/dominic-cummings-press-conference-blog-predicted-covid-19-coronavirus).

### Science in the Public Arena

The usual strategies and tactics needed to be jettisoned and in stepped the scientists to share the terrain of legitimacy. Here was a heterogenous body of scientists who became homogenous as reference to “the science” increased in public frequency. No longer about the narrow constitution of the economy and its potential and rhetorical allusions to a return to national sovereignty, this crisis was immediate and about health and then, health and the economy.

The Government can decide, in the face of an emergency response, when to set up the Scientific Advisory Group for Emergencies (SAGE). Its aim is to provide scientific and technical advice to support decisions in the Civil Contingencies Committee (COBR). In terms of the provision of independent scientific advice, overt governmental control was not an option, but a “presence” in scientific discussions became a second-best scenario. Again, this was exposed and calls followed for the Prime Minister’s then Chief Political Advisor to remove himself from SAGE and for their deliberations to be more transparent to the public (https://theconversation.com/dominic-cummings-and-sage-advisory-groups-veil-of-secrecy-has-to-be-lifted-137228).

In these circumstances the tactic to characterise scientists as experts seeking to legislate over what is possible and even desirable for the ways of life of the population could not play. Normally, once in the public sphere, the credibility of the expert becomes the same as the non-expert as the content of the knowledge produced is translated directly into its consequences for society. Most scientists recognise this dynamic. In view of the urgency and relevance of scientific insights for tackling the pandemic, the relations between credibility and applicability were short-circuited. In public policy terms their knowledge was compelling not just because it was useful, but because its visibility made it difficult to ignore (Mulgan 2009). With the questioning of expertise suspended, the realm of deliberation was informed by a shifting terrain of insights that sought to find patterns in emerging data and inform political decision-making beyond the usual sloganeering and narrow pre-occupations.

The scientific orientation was driven less by a search for certainty, but the value in understanding the desirability of various courses of action based upon emerging evidence. As Peter Piot, a microbiologist and Director of the London School of Hygiene and Tropical Medicine put it in reflecting on his own experiences of Covid-19: “The more we learn about the coronavirus, the more questions arise. We are learning while we are sailing” (https://www.sciencemag.org/news/2020/05/finally-virus-got-me-scientist-who-fought-ebola-and-hiv-reflects-facing-death-covid-19). Such a pragmatic orientation would expose tensions in institutional politics between the quality of deliberation and democratic decision-making (Outhwaite 2021). Avoidance of public, political accountability would inform a political-scientific dynamic as the pandemic evolved. We witnessed the creation of a public platform for science. In televised briefings the focus was upon a combination of scientific knowledge and political judgement. If the universal of science as the exercise of doubt through a preparedness to be exposed to falsification had been exploited by politicians and their technicians who felt no such compunction in pursing their certainties, it was now in a public space informed by a public need for consistent and clear political judgement.

The situation required that scientists act politically in the sense of achieving a consensus between their deliberations and advice and political decisions. As the Chief Medical Advisor put it in evidence to a House of Commons Committee: “I think what SAGE has to do is to try to take complex science and bring it to a position where we say, ‘This is the consensus view of where we are now, but we are clear about the function and purposes of argument.’ What I think is not helpful is to say, ‘Here are several different views,’ and ask somebody who is less knowledgeable to bring these together and come to a single view. In SAGE, we try to come up with a consensus view, but we are always clear and open about how we arrive at that” (https://publications.parliament.uk/pa/cm5801/cmselect/cmsctech/correspondence/200518-Chair-to-Prime-Minister-re-COVID-19-pandemic-some-lessons-learned-so-far.pdf). However, despite such assurances, concern about the lack of transparency and political interference in SAGE led David King, former Chief Scientific Advisor, to set up an independent SAGE whose deliberations were publicly available (https://www.independent.co.uk/news/uk/politics/coronavirus-sage-dominic-cummings-david-king-a9496546.html).

Steve Fuller’s characterisation of scientists as the “unelected masters of what remains unknown about us to ourselves” (2018: 45) carried with them the weight of public legitimacy in the pandemic. A simple collapse of the political and scientific through exploiting differences or condemning its applicability in the name of the market, would meet bodies of expert opinion who were regarded as doing their best in difficult circumstances. Although differences existed in scientific opinion with respect to modelling and alleviation of the effects of Covid-19 whilst “learning while sailing,” here were scientific forums for whom: “obstacles to rational persuasion produced by the lack of relevant knowledge, and the distractions of other business are eliminated, or much mitigated.” This rendered them: “much more effective in coming to conclusions than ordinary legislative bodies, whilst preserving the character of liberal persuasion within them” (Turner 2003).

In the face of these dynamics, we witnessed fluctuations in the desire for political leaders to share a platform with the public face of science. Increasing legitimacy for the generation of scientific knowledge was at odds with the scepticism and even contempt towards public institutions. A lack of clarity then followed with the emergence of new bodies. In the middle of the first phase of the pandemic, the Joint Biosecurity Centre was set up under the direction of a member of the National Cyber Security Centre, which is part of GCHQ (Government Communications Headquarters). The exact nature of the relations between this entity and SAGE and the role of evidence in informing public policy then became a matter of concern for the House of Commons inquiry into lessons learnt from the pandemic, as well as commentators in the British Medical Journal (see: https://committees.parliament.uk/work/657/coronavirus-lessons-learnt/ http://dx.doi.org/10.1136/bmj.m2874). Confusion and matters of blame displacement were exacerbated with the announcement that Public Health England, six months into the pandemic, would be merged with NHS test and trace and the Joint Biosecurity Centre (JBC) into the National Institute for Health Protection. In response to questions from a House of Commons committee, the director-general of the JBC made clear their differences from SAGE: “We are not an independent scientific body that has members per se. We are part of the civil service. We are staffed by civil servants and we report directly to the Secretary of State for Health and Social Care” (See: https://committees.parliament.uk/oralevidence/1085/pdf/). Public transparency appeared to be displaced through translation into Ministerial control.

As accountability and transparency became more clouded, the Government were taken into a terrain of public spending of approximately £300 billion by the end of 2020; serving to reinforce the reality of the interactions between politics and the economy (https://obr.uk/efo/economic-and-fiscal-outlook-november-2020/). In the process consultations with the public sector were informed by suspicion and by October 2020 an estimated £10 billion of public contracts were awarded to private companies in the absence of competitive tender (see: https://www.ft.com/content/7bf2fbdc-a26b-476e-a604-fac15ecfc222). The Health Secretary was later ruled to have breached his “legal obligations” in not publishing details of these contracts within a specific time frame (https://www.bbc.co.uk/news/uk-56125462). At the same time, the Coronavirus Act, granting emergency powers, led to the executive branch of Government acting without normal consultation with Parliament, thereby reducing its powers to hold them to account. The Act itself has been described as the “biggest restriction on civil liberties in a generation” (see: https://www.theguardian.com/commentisfree/2020/sep/29/coronavirus-act-liberties-powers-police-public-health-crisis).

### Space And Place: Context Finds a Voice

Along with the interplay between science and politics and issues of transparency and political accountability, the relations between space and place came under strain. Political differences emerged between the devolved administrations in the United Kingdom who spoke of the absence of communication and coordination with Westminster as impediments to the effectiveness of responses. COBR enabled the Prime Minister to meet with the First Ministers of Scotland, Wales and Northern Ireland. Yet it did not meet between May 10 and September 22, 2020, leaving a report on coordination and divergence in responses to the pandemic to conclude: “The use of intergovernmental fora has declined since May, reducing opportunities for the four governments to co-ordinate their approaches and manage the consequences of divergence” (Sargeant 2020). A reluctance to share centralised prerogatives with the devolved regions was also apparent within England.

It has been argued that the content of knowledge mobilized in neoliberal reforms is context-revising, not sensitive. It seeks to change and mould places in the image of abstract trans-local images (May and Perry, 2018). Impediments to this realisation are blockages to be removed. Sensitivity to social context entails political recognition of places and their representatives. Urgency for change provides no time for reflection or learning in the name of re-fashioning contexts for the future: “The final power would thus be less one of imagination than of anticipation, so much that to govern would *be* no more than to foresee, simulate, memorize the simulations” (Virilio 1986). With time and power aligned in these ways, context is not expected to speak back. That changed in the pandemic.

The introduction of eight elected mayors in England (London has had a mayor with differing powers since 2000) from 2017–2019, who are also chairs of a Combined Authority, introduced a layer of accountability for devolved powers from central Government. The terms of devolution provide for some flexibility, whilst election, as Rancière (2006) reminds us, is also a process of conferring legitimacy for subsequent political decisions. In the case of Combined Authorities this is informed by knowledge through acquaintance with the area, its dynamics and representation of its interests. As relations between time and place altered as the pandemic unfolded, the Government introduced a tier system. “Alert levels,” from medium through high to very high, were introduced and deployed to enable a judgement to be made between a “return to normal” measured against transmission rates, with varying degrees of financial support available depending on the level assigned; all of which took place against a backdrop of twenty two billion pounds of initial investment in a test and trace programme, with an additional allocation of fifteen billion over the next two years (https://committees.parliament.uk/committee/127/public-accounts-committee/news/150988/unimaginable-cost-of-test-trace-failed-to-deliver-central-promise-of-averting-another-lockdown/).

Questions over the test and trace programme and use of a tier system created a space for place-based judgement and mobilisation of local knowledge. This was bolstered by private contractors, paid to set up and implement a national test and trace system, not providing an effective process (see: https://www.theguardian.com/commentisfree/2020/nov/03/advice-schools-covid-infection-pupils-classrooms-test-and-trace). The result was an increased local involvement in contact tracing, utilising local expertise, to reach communities. Such practices challenged the centralising tendencies in English politics leading the Mayors of London and Manchester to write: “We are uniquely placed to help. As mayors our focus is exclusively on the city regions we run. But the Westminster Punch and Judy show struggles to relate to this more grown-up and pragmatic ‘place before party’ approach” (https://www.theguardian.com/commentisfree/2020/oct/25/mayors-are-a-force-for-good-and-its-time-johnson-recognised-that).

Political tensions increased as a proposed Government imposition of tier 3 upon Greater Manchester in October 2020 proved highly contentious. The Mayor of Greater Manchester argued that, should this take place, greater levels of support were needed for business. Politically, however, this was not about the levels of funding as such, with the Government invoking “fairness” in comparison to other tier 3 areas in the North of England, but about an English North-South divide in terms of leveling up of support, not down. That point was not lost on those Conservative MPs elected in 2019 from Northern England which resulted in differences within party ranks (https://theconversation.com/andy-burnhams-standoff-with-london-was-always-about-more-than-just-lockdown-money-148594). Tier 3 was ultimately introduced in Greater Manchester as the Mayor made it clear that it was not desirable to break the law.

It transpired that SAGE had earlier recommended a national “circuit breaker” to prevent the spread of the virus and avoid a higher number of deaths. With the Welsh Government introducing a 17-days virus “fire-break” and Scotland and Northern Ireland imposing their own preventative measures, the political terrain was exposed to regional contestation and bolstered by scientific recommendations concerning the need for a national response which did not discriminate on the grounds of place. As the Chief Medical Advisor put it in evidence to the House of Commons Select Committee: “the argument for strong regional variation in what we do is not terribly convincing” (https://publications.parliament.uk/pa/cm5801/cmselect/cmsctech/correspondence/200518-Chair-to-Prime-Minister-re-COVID-19-pandemic-some-lessons-learned-so-far.pdf). A three-week gap between the Government receiving the SAGE advice and the announcement was justified as being acceptable within the bounds of a “balanced view” (https://www.thetelegraphandargus.co.uk/news/18790627.government-ignored-sage-advice-lockdown-month-ago/).

Schisms between and within politics and science grew. Allegations were made that the Government were in the grip of scientists as unaccountable elites, along with the role of private companies and the pursuit of profitability in the process of science and the funding of the work of scientists themselves (https://www.thelancet.com/journals/lancet/article/PIIS0140-6736(20)32064-X/fulltext). As cases exceeded one million and predictions that the death rate could be twice that of the first lockdown, the English Government performed a ‘strategic’ U-turn and England went into a four-week lockdown at the end of October, with a third following in January 2021.

## The Redemptive Hits Limits

These dynamics can be further understood by taking the earlier discussions concerning pre-pandemic conditions and placing them within the literature on populist politics. Although populism can be a vague term with writers contributing to its clarification (Laclau 2007; Mouffe 2018) Canovan (1999) draws on the work of Oakeshott (1996) to develop her ideas. Oakeshott contrasted the “politics of faith” with the “politics of scepticism.” The former is concerned with the pursuit of salvation and requires for its energy the “mobilization of popular enthusiasm” and “the quest of power to accomplish it” (Canovan 1999). The latter, on the other hand, is suspicious of such motives, reduces its expectations of what politics can achieve and is concerned with order and the reduction of conflict: “For this style of politics, the rule of law is crucial” (Canovan 1999). Oakeshott regarded these two styles of politics as inseparable.

Canovan does not consider these terms sufficiently clear. Instead, she uses the terms “pragmatic” and “redemptive.” Whilst seemingly opposed to each other she argues they are also interdependent. Democracy has a redemptive vision “kin to the family of modern ideologies that promise salvation through politics.” Its pragmatic face, however, is that it is a form of government which is “a way of running what is always one particular polity amongst others in a complex world” (Canovan 1999). In the gap between them, populism can appear: “one could caricature democracy’s pragmatic face with the slogan, ‘ballots, not bullets’, or (in more academic terms) as ‘a system of processing conflicts without killing one another’. A corresponding caricature of the redemptive face might be ‘*vox populi vox dei’*, or ‘government of the people, by the people, for the people’” (1999: 9–10. Original Italics).

The redemptive requires the pragmatic for its realisation through the deployment of rules and practices to settle conflicts peacefully. If the redemptive is in ascendancy it emphasises the idea that the people are the source of legitimate authority which becomes apparent only when they take charge of their own lives. Such a realisation does not necessitate democracy and looking around the world, it is one form of government among others for running affairs. Functioning democracies, however, require institutions not only to limit power, but channel it and render it more effective. If the redemptive takes precedence we find: “a strong anti-institutional impulse: the romantic impulse to directness, spontaneity and the overcoming of alienation” (Canovan 1999). As we have seen, for the current English Government it aligns with an attitude that runs from suspicion to contempt for public institutions that need transformation in the abstract image of a market apparently separate from the realm of politics which is increasingly emptied of public accountability.

This form of redemptive politics takes society down a path of limited capability when faced with the collective risk of a pandemic. Fluctuations between strategies of mitigation and suppression of the pandemic in the face of the need for overt Government-led interventions become more likely. Suspicion of public service, in comparison to the assumed efficiencies of the private sector, become internal to a regime charged with responsibility for tackling and alleviating its consequences. Although the process tempered this right-wing populism, it informed an ambiguity in the coherence and consistency of political judgements. We witnessed a preference for awards to private companies, despite a huge variation in their capabilities to deliver; an absence of preparedness to discuss and coordinate with the devolved administrations, as well as with English city and regional political representatives.

Right-wing populism was riding high before the pandemic, particularly with the promise of a return to popular sovereignty following the outcome of the 2016 referendum to leave the European Union. Political rhetoric referred to external threats to liberty and sovereignty whilst, at the same time, exhibiting tendencies to regard democracy as embodying the “impurity of politics” and a “challenging of governments claims to embody the sole principle of public life” (Rancière 2006). This opened up a political space where we find leaders as “vivid individuals who can make politics personal and immediate instead of being remote and bureaucratic. In this context, amateurism and lack of political experience actually become recommendations” (Canovan 1999). A lack of experience and amateurism would not, however, be appropriate for tackling a societal pandemic. Limits were exposed and the Government were taken into uncomfortable terrain: “When states are targeting asset prices, providing wholesale bailouts to private corporations and buying up substantial portions of their own debt, it becomes far harder to argue that interventions to promote the public good are undesirable because they might disrupt the operation of the market mechanism” (Blakeley 2020).

Attempts to dismantle the boundary of the political through a focus on the economics of things, not people, could not work. People, their families and communities were profoundly affected by Covid-19 which ran through the fault lines of socio-economic inequality. A pragmatic response was needed from political leaders. Normally, they seek to configure a heterogenous population through a focus on micro demands in the marketplace. The pandemic brought the present into sharp focus and the response had to be much more than the creation of a playing field for the few over the needs of the many. The population had moved from heterogenous spectators. A need arose for good judgement and transparency in political decision-making which is so often displaced by a media fuelled theatre of scandal. That induces a passivity among citizens informed by grievance and complaint within “spectator democracy’ (Han 2017). The pandemic created a homogenous expectation to act on an internal matter with very high, collective risks. That translated into active expectation of political leaders which exposed the limits of their usual actions.

These tensions informed how science and politics interacted. Steve Fuller’s discussion of anticipatory and precipitatory governance, although referring to innovation, is helpful here. The former is concerned with a precautionary principle such that those things which are likely to create more harm than good would not be pursued. The latter, on the other hand: “operates on the assumption that some harm will be done, no matter what course of action is taken, and that the task is to derive the most good from it” (2018: 175). In this latter course we find a calculation that harm may be done in the short-term, but over time a better outcome will emerge. That is the preferred form for those who emphaise disruption in the name of a future in which the end justifies the means.

Politicians of this persuasion and their associated technicians of transformation and turmoil often follow the command of “no pain, no gain” (Fuller 2018). For this reason, the activation of public legitimacy ascribed to the practices of a precautionary science was met first by denial and then, varying degrees of incorporation. The usual tactics to supress knowledge circulating in the domain of public accountability where anxiety, fear and expectation were rife, could not play. Concerns and consequences were both generalized and personal and in search of answers not reducible to market preference. That created a powerful confluence not amenable to being met with the usual tactics. A realm opened-up where public expectation focused on the production of knowledge informed by a precautionary principle as a challenge to usual epistemic relations that work to: “insulate themselves from critical challenge by distorting the space of reasons and presenting relations of rule or domination as ‘natural’ (unalterable), ‘God-given’, or in some way falsely, as sufficiently justified” (Forst 2014).

Those dynamics informed the shifting boundaries between scientific advice and political judgement. Science had a public face and that informed judgement of political decision making. However, scientific practices themselves were also challenged. After all, if they do not understand this political climate, a reflexive blindness to the conditions informing practice will emerge. If you are “learning to fly whilst in the plane” and do so whilst assuming high degrees of “epistemic impermeability” (May with Perry 2011) are in place that assume a simple separation between knowledge production and reception, you will not remain in the air for long. Transparency in these relations is important and its absence in SAGE led to an independent group being set up in the face of a democratic lag in which normal parliamentary scrutiny was suspended. Concerns with scientific consensus, particularly when laws are being deployed to circumvent normal democratic process, can be problematic when dealing with rapidly changing situations: “Assertions about scientific consensus circumvent debate within the scientific community and with others inside and outside academia with relevant expertise; rapid imposition of laws or regulations precludes mechanisms of democratic control that would usually be expected before such major policy interventions, such as parliamentary debates or impact assessments” (Martin et al., 2020).

Scientific practices had much to learn and needed to adapt in this fluid climate. As the pandemic evolved, viewpoints from different disciplines were required. A scientific hierarchy was at play during the early stage of the pandemic, as is common in situations requiring interdisciplinary responses (see Callard and Fitzgerald, 2017). “Behavioural scientists” became involved. This is a generic term that covers the “othering” of disciplines by those in positions to make these judgements. Social factors were key to understanding and we moved from what seemed an initial reluctance to recognise these factors, to those with an understanding of their dynamics playing an increasing role in deliberations over time. The issue remains, however, as to whether they engage in the generation of insights on an equal basis or are expected to examine the implications for the public of the application of insights already placed in the realm of unproblematic justification (May and Perry, 2022). Whilst sufficient evidence on such issues is yet to emerge, early studies suggest that such integration was not apparent and whilst the urgency of the situation may explain some outcomes, it does not preclude more imaginative responses which incorporate a wider variety of perspectives (Martin et al., 2020).

If the pandemic has taught us anything about the relations between science and society, it is the need for more sustainable “civic epistemologies” (Jasanoff 2012). What lies at the heart of democracies, exacerbated by this right-wing populism, is how particular experts are enrolled into the apparatus of formal government bodies and how such knowledge is produced and interpreted and deployed. Sheila Jasanoff refers to this as living in an “Expert Raj” in which such processes: “are as opaque to ordinary citizens as the self-legitimating claims of rulers in distant metropoles were to colonial subjects living in the peripheries of empire” (2012: 11). A way to approach this is to examine events, such as the pandemic, where the: “principles underlying trust in government by experts are exposed to public scrutiny” (2012: 11). That requires degrees of transparency which, as we have seen, have not been sufficiently evident and are also being eroded.

When it comes to an understanding of this context from health scientists, it is argued that in Britain we find “a tradition of respect for evidence” but “data tend to be used as weapons in political debate rather than for reasoned argument” (Marmot 2015). In characterising the issue in this way and in dealing with matters of public health, Michael Marmot seeks to maintain “the fiction that I am not political” (2015: 342). Despite this, his conclusions are clear: “disempowerment, material, psychosocial and political, damages health and creates health inequities” (2015: 346). Similar observations are apparent in recent observations on the pandemic whereby it is the form of capitalism pursued by political leaders that has ripped into the ‘social fabric’ of society (Horton 2020). We find a clear ambivalence here regarding the boundaries between the political and scientific which, as we have seen, have sought to be reconfigured in the name of narrow notions of the economy, separate from society. Their maintenance to ensure scientific credibility is important, but they move over time and in the current era political distortion is in the ascendancy. To maintain such credibility, particularly in the face of how the pandemic has exacerbated existing, deep rooted inequalities, science must understand that and build it into its practices, or its risks reproducing this situation.

## Summary

A virus of precarity brought about by the actions described above, found itself confronted by coronavirus which tracked through the fault lines of socio-economic inequality. A redemptive distaste for democracy and the public institutions which maintain its pragmatic dimensions informed Government responses to the pandemic. Scientific practice operated on a precautionary basis at odds with the tendency towards precipitatory governance. The consequences in ill-health and death in the United Kingdom are evident and whilst a public inquiry into the handling of the pandemic has been announced for Spring 2022, its remit and date of reporting is yet to be determined (https://www.bbc.co.uk/news/uk-57088314).

We have seen calls to “go back” to the economy and exercise the precautionary principle via allusion to “following the evidence”; particularly when faced with political dissent from within the ranks of the Conservative Party. Overall, the desire to move beyond the current situation is understandable. At the time of writing, we have seen nearly one hundred and thirty thousand deaths; continuing symptoms of the effects of long-Covid; rising mental health issues; effects on education and learning among young people; increased rates of unemployment; higher rates of domestic violence; delayed operations and amplification of already existing huge inequalities in health and income and levels of public debt not seen since the two World Wars. As it stands, will the levels of support provided for business be given over to the public sector and those in need in the future? The National Health Service had considerable public support, but will the funding for this institution and its workers, having repeatedly proved themselves to society in such awful circumstances, be exempt from the impulses charted here? We are led by those who know what they dislike, born of their selected experiences transposed into a generality through the creation of socio-economic conditions that have dire consequences for most. It is accompanied by a refusal to dwell on history and how it forges the present in favour of a focus upon imaginary futures of limitless potential.

Despite attempts to break the boundaries between the economy and well, just about everything else, expressions of general political conflict during the pandemic occurred. We witnessed this in Black Lives Matter. That brought to attention not only to a critique of the political speaking as if it were a universal, but a demand for social justice. In the process the apparent solidities of the past, constituted in statues of the venerable, were exposed as built upon violence and exploitation (https://www.bbc.co.uk/news/av/uk-52954994). Running through to current times, it became a history of the present exposed as the struggle between memory and forgetting. No wonder that the representatives of the political ideology charted here want history to be taught as the reciting of dates, celebration of Monarchs and overall, rendering its study as a nostalgic revere of yesteryear. Whilst defending the right to dissent, these protests were described by the Home Secretary as “dreadful” and actions by the London Mayor to form a commission into diversity were characterised by the Leader of the House of Commons as a “loony left-wing wheeze” (https://www.theguardian.com/politics/2021/feb/12/priti-patel-hits-out-at-dreadful-black-lives-matters-protests).

The analysis presented here is not complacent concerning the public institutions of democracy. As Black Lives Matter demonstrated: “The crisis ushered in by coronavirus has accelerated the need to find this common ground between the defenders of institutional norms and those who agitate for economic justice” (Davies 2020). Whilst this Government seeks to dismantle them, for those who seek a more equal society, they need reform. Nevertheless, in the process we can easily forget, albeit flawed, how they came about: that is, to address the limitations of a society seen as indifferent to human need. In the meantime, political space is occupied by visions and practices which, not so long ago, would have been regarded as extreme. Knowledge has become a tool to manipulate, distract and persuade. We have not entered a period of post-truth. We live in an era in which knowledge is selected and deployed by those whose visions are myopic and for which society is paying a very high price.

This is not a politics seeking to recognise differences in the name of greater understanding and tolerance and respect, but one which atomizes society. It mobilises prejudice and mistrust to suit a desire for power to run uninhibited through the body politic. The legitimacy that holds such power in check is at an all-time low and with that political accountability. Its forward march has been tempered by the pandemic. The call for a “return to freedom” is part of current political rhetoric, but so too is the desire of the population to return to a greater sense of normality. Despite the death toll, public focus is now upon vaccination rates and the hope for a return to normal. Once again, time and power interact and with that the propensity for forgetting to triumph over memory. Relieved of fear through the desire to return to normal, the issue is not only effective control of the pandemic, but that the problems which result from this politics will persist and learning will, once again, depart.

## Data Availability

The original contributions presented in the study are included in the article/supplementary material, further inquiries can be directed to the corresponding author.
